# The Histone Acetyltransferase Gcn5 Regulates ncRNA-*ICR1* and *FLO11* Expression during Pseudohyphal Development in *Saccharomyces cerevisiae*

**DOI:** 10.1155/2015/284692

**Published:** 2015-04-02

**Authors:** Long-Chi Wang, Fernando Montalvo-Munoz, Yuan-Chan Tsai, Chung-Yi Liang, Chun-Chuan Chang, Wan-Sheng Lo

**Affiliations:** ^1^Biotechnology Center, National Chung Hsing University, Taichung 402, Taiwan; ^2^Department of Life Sciences, National Chung Hsing University, Taichung 402, Taiwan; ^3^Institute of Plant and Microbial Biology, Academia Sinica, Taipei 115, Taiwan

## Abstract

Filamentous growth is one of the key features of pathogenic fungi during the early infectious phase. The pseudohyphal development of yeast *Saccharomyces cerevisiae* shares similar characteristics with hyphae elongation in pathogenic fungi. The expression of *FLO11* is essential for adhesive growth and filament formation in yeast and is governed by a multilayered transcriptional network. Here we discovered a role for the histone acetyltransferase general control nonderepressible 5 (Gcn5) in regulating *FLO11*-mediated pseudohyphal growth. The expression patterns of *FLO11* were distinct in haploid and diploid yeast under amino acid starvation induced by 3-amino-1,2,4-triazole (3AT). In diploids, *FLO11* expression was substantially induced at a very early stage of pseudohyphal development and decreased quickly, but in haploids, it was gradually induced. Furthermore, the transcription factor Gcn4 was recruited to the Sfl1-Flo8 toggle sites at the *FLO11* promoter under 3AT treatment. Moreover, the histone acetylase activity of Gcn5 was required for *FLO11* induction. Finally, Gcn5 functioned as a negative regulator of the noncoding RNA *ICR1*, which is known to suppress *FLO11* expression. Gcn5 plays an important role in the regulatory network of *FLO11* expression via Gcn4 by downregulating *ICR1* expression, which derepresses *FLO11* for promoting pseudohyphal development.

## 1. Introduction

Fungi can alternate their cellular morphology between unicellular yeast and multicellular hyphae forms in response to environmental stimuli, a process known as dimorphic switching. This phenomenon is intimately linked to pathogens of animals or plants and their pathogenicity [1, 2]. The pathogenic fungus* Candida albicans* switches from the usual unicellular yeast-like form to a multicellular invasive-filamentous form when it infects host cells [3]. The budding yeast* Saccharomyces cerevisiae* can switch between different morphological forms under various stress conditions. By doing so, yeast features differential growth modes according to adaptive needs to confer cell protection and enhance dissemination and substrate colonization [4, 5].

In* S. cerevisiae*, cell-cell and cell-surface adhesion are required for many developmental processes including mating [6], haploid invasive growth [7, 8], diploid pseudohyphal development [9, 10], and biofilm formation [11, 12]. Each of these events is initiated by distinct signals that are coupled to the expression of specific cell surface proteins by corresponding signaling pathways (for reviews, see [13, 14]). For example, in response to nutrient stress such as nitrogen starvation, budding yeast develops two types of cell forms: adhesive/invasive growth for haploids and pseudohyphae for diploids [8, 9]. For both types of cell growth, yeast features increased cell length, change in polarity, and augmented cell-cell adhesion; as in pathogens, this kind of morphological development is essential for host-cell attachment, virulence, and tissue invasiveness [15].

The flocculin protein, Flo11, plays a central role in pseudohyphal growth and biofilm formation [11, 16–20]. In yeast cells with the* FLO11* deleted (*flo11*Δ), diploids do not form pseudohyphae and haploids lose agar invasiveness [17]. Unlike most genes in yeast, the upstream region and promoter of* FLO11* consist of an unusually long sequence spanning more than 3 kb and harboring binding sites for transcription factors involved in mitogen-activated protein kinase (MAPK) and protein kinase A (PKA) signaling pathways and the general control nonderepressible (GCN) response pathway [16, 21–26]. Genetic analyses and *β*-galactosidase reporter assays demonstrated that several transcriptional factors, including repressors and activators, coordinately regulate* FLO11* expression [24, 27–30]. Ste12 and Tec1 are nuclear transcription factors in the MAPK signaling cascade activating* FLO11* expression under nitrogen starvation [31]. The protein kinase Tpk2 in the cAMP-dependent signaling pathway is required for transcriptional activation of* FLO11* expression mediated by the transcriptional factors Flo8 and Gcn4 [21, 25, 26, 32]. GCN pathway is involved in both morphogenesis and biofilm formation in response to amino acid deficiency. The sensor kinase Gcn2 regulates expression of the transcriptional activator Gcn4 under conditions of amino acid deprivation, which consequently activates* FLO11* expression and pseudohyphal growth [21, 33].

Chromatin is composed of histone octamers (histones H2A, H2B, H3, and H4) that associate with DNA in nucleosomes. These structures can both repress and activate transcription and other genomic processes, such as cell progression, DNA replication, recombination, and repair, depending on the positioning of nucleosomes relative to binding sites for activators and repressors [34, 35]. A key event in the regulation of eukaryotic gene expression is the posttranslational modification of nucleosomal histones, which converts chromosomes into transcriptionally active or inactive chromatin. A gene-specific and time-dependent order of events is believed to link chromatin structure modifications and transcription activation. Chromatin-modifying enzymes mark histone residues and change nucleosome conformation, which allows the transcriptional machinery to transcribe or repress genes [36, 37].

Many epigenetically regulated genes including* FLO11* can be considered transcriptional switches because they have two inheritable expression states, “ON” and “OFF” [38].* FLO11* is generally silent in the yeast form unless transcriptional activation is induced by various environmental stresses for subsequent promotion of invasive growth in haploid cells and filamentous growth in diploid yeast.* FLO11* expression is repressed by the histone deacetylase (HDAC) Hda1, and importantly the Hda1-mediated gene silencing of several* FLO* genes depends on subtelomeric chromatin position [39]. However, a histone deacetylase complex containing Rpd3L plays a seemly paradoxical role in activating instead of repressing* FLO11* expression [40]. In addition, the chromatin-remodeling complexes Swi/Snf and Rsc can regulate* FLO11* expression [41, 42].

Two long noncoding RNAs (ncRNAs),* ICR1* (interfering Crick RNA) and* PWR1* (promoting Watson RNA), are transcribed by RNA polymerase II in an opposite direction within an overlapped sequence at the upstream promoter region of* FLO11* [43, 44]. Rpd3L acts with two transcription factors, Flo8 (an activator) and Sfl1 (a repressor), to regulate the mutually exclusive transcription of* PWR1* and* ICR1*. Sfl1 suppresses* PWR1* transcription by competing with Flo8 to bind the “toggle” sites at the* PWR1* promoter and then induces* ICR1* expression, which then suppresses* FLO11* transcription via a promoter exclusion mechanism. When Rpd3L binds to the* FLO11* promoter, Sfl1 is excluded from the* PWR1* promoter, which leads to Flo8 associating with the toggle sites to activate* PWR1* and concomitantly blocks* ICR1* transcription. Consequently, Rpd3L and Flo8 activate* FLO11* expression by regulating the reciprocal transcription of a pair of cis-interfering ncRNAs [43, 45].

Gcn5 is a histone acetyltransferase (HAT) that functions as a coactivator in transcriptional regulation and mainly contributes to transcriptional activation and substrate specificity combined with other histone modifiers [46, 47]. Gcn5 modifies several of the aminoterminal lysine residues of histones with acetylation and is the catalytic subunit for three chromatin-modifying complexes, ADA, SAGA, and SLIK/SALSA, to regulate a wide arrange of genes both positively and negatively [48, 49]. The histone acetylation activity of Gcn5 is required for Gcn4 to act as an efficient transcription factor at gene promoters [50]. Gcn4 recruits Gcn5 and its multisubunit complexes to the promoters of a specific subset of genes, such as* HIS3*, for transcriptional activation [51, 52]. Although Gcn5 was found involved in* FLO11* expression under amino acid starvation, such regulation was observed only in haploid strains [42].

The mechanism to control the precisely regulated switch from repression to activation of* FLO11* represents a critical question in yeast dimorphism. In this study, we demonstrate that Gcn4 and Gcn5 are new components in controlling* FLO11* expression and pseudohyphal development in diploid yeast under amino acid starvation by regulating ncRNA* ICR1* transcription at the promoter of* FLO11*.

## 2. Materials and Methods

### 2.1. Plasmids and Yeast Strains

The yeast strains used in this study were all in a* S. cerevisiae* Σ1278b genetic background and are described in Table 1. Standard yeast culture medium was prepared as described previously for invasive growth in YPD (Bacto Peptone; 4% Bacto Peptone, 2% yeast extract, 2% peptone, and 2% glucose) and for filamentous growth in SLAD (synthetic medium with a low concentration of ammonium (6.7 g/L yeast nitrogen base without amino acids and ammonium sulfate, 2% glucose, and 2% washed Bacto Agar)) [8, 9]. Amino acid starvation medium was prepared by supplementing minimal media (1.7 g/L yeast nitrogen base without amino acids and ammonium sulfate and 2% glucose) with 10 mM 3-amino-1,2,4-triazole (3AT) [47]. Yeast transformation involved the lithium acetate method and all yeast manipulation followed standard methods [53, 54]. The plasmids carrying* MATa1* (pRS315-*MATa1*) or pRS414-*MATαAT* in haploids were constructed as described previously to generate pseudodiploid strains [55]. The construction of Gcn5 mutants was performed as described previously [47]. The vector pRS316 was used to generate a wild type* GCN5* (pRS316+GCN5-WT) and the single amino acid substitution of Glu to Gln (E173Q; pRS316-yGCN5-E173Q) representing functional Gcn5 and a catalytically dead mutant of* GCN5,* respectively.

### 2.2. Invasive Growth and Pseudohyphal Formation Assays

Measurement of invasive growth of yeast cells was as described [55]. Briefly, a 3 mL YPD liquid culture was grown until it reached OD_600_ 0.8–1 and then 5 *μ*L was plated into two YPD agar plates and incubated for 4 d at 30°C. One of the plates was used for measuring “total growth” and the other for “invasive growth” remaining on the agar media after two washes as described. Total growth was measured by excision of the yeast cells together with agar and liquification and quantification of cells by OD_600_. The second plate was washed with 9 mL distilled water by use of a microtiter plate shaker at 5 rpm for 5 min each time. The cell number was measured as described for the total growth. The analysis of pseudohyphal filament formation was as described [9]. Yeast cells were streaked on SLAD plates to score phenotype after growth at 30°C for 6 d. Plates were photographed by use of a digital camera attached to a stereomicroscope (ZEISS) and the number of colonies forming filaments was counted.

### 2.3. RNA Preparation, Reverse Transcription, and Real-Time qPCR

Total RNA was extracted from yeast cells using a total RNA Mini Kit (Geneaid) as described [56, 57]. To remove potential contamination of genomic DNA, total purified RNA was incubated with RQ1 (Promega) DNase at 37°C for 40 min and then inactivated at 65°C for 10 min. Reverse transcription involved a cDNA synthesis kit (Epicentre) in an RNase-free environment. RNA underwent initial incubation at 65°C for 5 min with oligo-dT. Then, the reaction mixture including buffer, dNTPs, DTT, ribonuclease inhibitor, and MMLV reverse transcriptase was incubated at 37°C for 60 min and then inactivated at 85°C for 5 min. cDNA prepared from triplicate biological samples was used for PCR analysis. Real-time quantitative PCR (qPCR) involved use of Stratagene Mx3000p (Agilent Technologies) and Kapa SYBR Fast Universal qPCR Kit (Kapa Biosystems). The primers used in this study are available upon request.

### 2.4. Chromatin-Immunoprecipitation (ChIP) Assay

ChIP assay was performed as described previously [56, 57]. Briefly, the haploid and diploid Gcn4-Flag cells were fixed with formaldehyde for 2 h. To maintain a proper size of fragmented chromatin, we optimized the sonication parameters to shear DNA to an average size of about 200 bp corresponding to 1 or 2 nucleosomes. Chromatin solution containing 1 mg whole cell extract was immunoprecipitated (IP) with anti-Flag M2 (Sigma-Aldrich) antibody and purified with protein G sepharose (Millipore). The precipitated DNA was analyzed by real-time qPCR. The signal for each gene primer pair in the immunoprecipitation was normalized to that of the input and then divided by the control vector to determine the fold change. Quantification of data, indicated by mean ± SD, was based on the number of independent biological and/or experimental replicates described in the figure legends.

## 3. Results and Discussion

### 3.1. Gcn5 Is Involved in* FLO11* Expression and Pseudohyphal Development in* S. cerevisiae*

**Figure 1:**
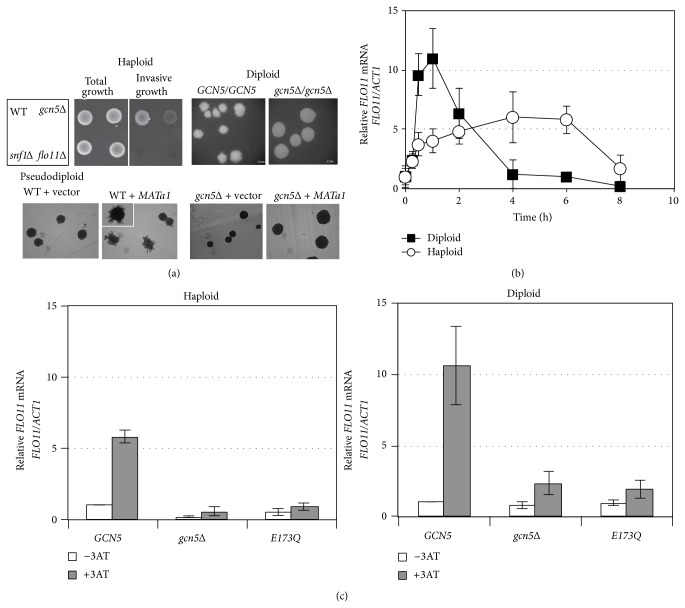
Gcn5 is involved in pseudohyphal development and* FLO11* expression. (a) Gcn5 is required for invasive growth in haploids and filamentous growth in diploids. Haploid cells of wild type (WT) yeast and deletion mutants (*gcn5*Δ,* snf1*Δ, and* flo11*Δ) were grown on YPD agar medium for 4 d at 30°C before analyzing invasive growth (haploid, top left panel). Cells grown on agar surface before (total growth) and remaining after being rinsed with water (invasive growth) are shown. The WT (*GCN5/GCN5*) and homozygous* gcn5*Δ diploids (*gcn5*Δ*/gcn5*Δ) were grown on nitrogen starvation agar medium (SLAD) for 6 d at 30°C before filamentous growth was recorded (diploid, top right panel). Shows filamentous growth of colonies on SLAD agar medium for diploid WT (*GCN5* + vector) and* gcn5*Δ (*gcn5*Δ + vector) and pseudodiploid WT (*GCN5* +* Mat a1*) and* gcn5*Δ (*gcn5*Δ +* Mat a1*) (pseudodiploid, bottom panel). Scale bar: 0.5 mm. (b) The expression patterns of* FLO11* in haploid and diploid yeast in response to 3AT. The isogeneic haploid and diploid strains were grown at 30°C in minimal medium to 0.8 OD_600_ (time 0) and induced with 10 mM 3AT. Total RNA was prepared from samples collected at the times indicated. Quantitative RT-PCR (RT-qPCR) analysis of the mRNA levels of* FLO11* normalized to that of* ACT1*. (c) The histone acetyltransferase activity of Gcn5 is required for induced* FLO11* expression by 3AT. Total RNA was prepared from samples collected at 2 h (haploid) or 1 h (diploid) after the addition of 3AT. The* gcn5* deletion haploid (left panel) or diploid (right panel) strains carrying a wild type* GCN5* (*GCN5*), vector (*gcn5*Δ), or a catalytic dead mutant (E173Q) were grown in repressed (−3AT) or induced condition (+3AT, 10 mM). RT-qPCR analysis of the mRNA levels of* FLO11* normalized to that of* ACT1* and compared with the WT (*GCN5*) without 3AT. Data are mean ± SD from 3 biological repeats ((b)-(c)).

To investigate the role of histone modifiers in pseudohyphal development by controlling* FLO11* gene expression in yeast* S. cerevisiae*, we examined the phenotype of haploid and diploid strains with deletions of* GCN5* (*gcn5*Δ) and* SNF1* (*snf1*Δ), which encode histone acetyltransferases and histone kinase, respectively. In haploids, wild type (WT), but not* gcn5*Δ,* snf1*Δ, and* flo11*Δ, remained on agar plate medium after being washed with water, so* GCN5* and* SNF1* are required for invasive growth (Figure 1(a)). In diploids, the homozygous mutant* gcn5*Δ/*gcn5*Δ did not develop the pseudohyphae shown in the WT (Figure 1(a)). In addition, we constructed a pseudodiploid by introducing a single* MATa1* plasmid to the *α* haploids [55] and found that the pseudohyphal development was suppressed in* gcn5*Δ (Figure 1(a)). These observations convincingly support the essential role of the histone acetyltransferase Gcn5 in haploid invasive and diploid filamentous growth.

*FLO11* is required for both haploid invasive and diploid filamentous growth, generally considered to be mediated by distinct pathways [17, 39]. We examined the induction of* FLO11* expression in both haploids and diploids under an amino acid starvation condition induced by 3AT. In a time course experiment, the induction patterns of* FLO11* mRNA expression in diploids and haploids differed. In haploids,* FLO11* transcription was activated gradually upon 3AT treatment, with approximately 8-fold enhancement 4 h after induction followed by a decrease after 6 h (Figure 1(b)). In diploids,* FLO11* expression was quickly induced by 10-fold in <30 min and reached approximately 12-fold enhancement by 1 h and then was reduced to the basal level at 4 h after 3AT induction (Figure 1(b)). This was an unexpected observation, because* FLO11* was previously not considered to be expressed in diploids and tetraploids [58, 59] and the induction of* FLO11* by nitrogen starvation was found much lower in diploids than haploids [21]. This discrepancy may result from different time courses in sampling.

### 3.2. HAT Activity of Gcn5 Is Required for* FLO11* Expression Induced by Nitrogen Starvation in Dimorphic Growth

Gcn5 was found to regulate* FLO11* expression under amino acid starvation in haploid yeast [42]. However, whether Gcn5-mediated* FLO11* expression is involved in dimorphic growth was unclear. We previously showed that a mutation (E173Q) in the catalytic domain of Gcn5 disrupted its HAT activity and resulted in poor growth under nitrogen starvation [47]. To ascertain whether Gcn5 and/or its HAT activity is essential for transcriptional regulation of* FLO11* with amino acid starvation, we first measured* FLO11* transcripts in deletion (*gcn5*Δ) and catalytically dead (E173Q) Gcn5 strains with and without 3AT. 3AT-induced* FLO11* expression was significantly impaired in* gcn5*Δ in both haploids and diploids at 2 and 1 h after treatment, respectively (Figure 1(c)). Furthermore, HAT activity of Gcn5 was critically important for the full induction of* FLO11* by 3AT (Figure 1(c)). Therefore, Gcn5 as an HAT is required for transcriptional activation of* FLO11* in both haploids and diploids under amino acid starvation.

**Figure 2:**
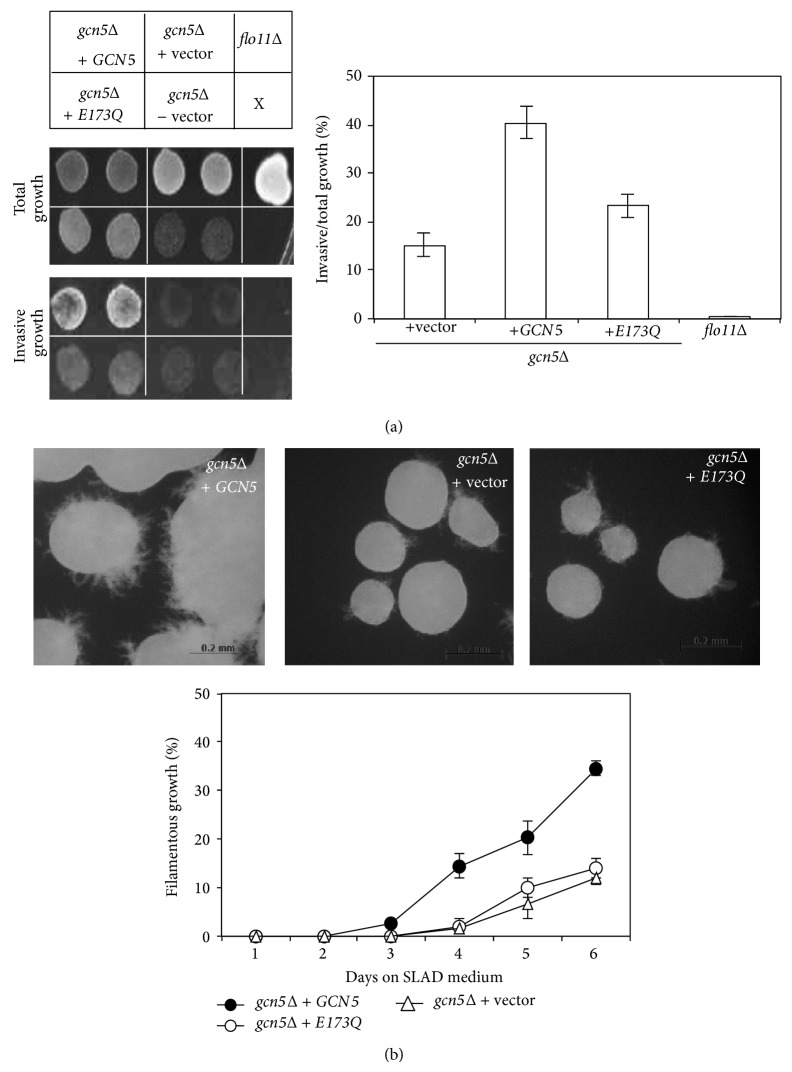
The histone acetyltransferase activity of Gcn5 is required for pseudohyphal development. (a) The histone acetyltransferase activity of Gcn5 is required for haploid invasive growth. The* gcn5* deletion haploid (*gcn5*Δ + vector) strain carrying a wild type* GCN5* (*gcn5*Δ +* GCN5*) or a catalytic dead Gcn5 (*gcn5*Δ + E173Q) was grown on YPD agar plate for 4 d at 30°C before invasive growth was scored. The* flo11*Δ was used as a control for complete loss of invasive growth. The quantification of invasiveness in each strain was measured by cells remaining on agar before (total growth) and after being washed (invasive growth) and is shown on the right. Data are mean ± SD from 5 measurements in 10 biological repeats. (b) Histone acetyltransferase activity of Gcn5 is required for diploid filamentous growth. The indicated diploid strains were grown on nitrogen starvation agar medium (SLAD) at 30°C and the filamentous growth of colonies was recorded daily for 6 d. The quantification of filament formation was described in Section 2. Data are mean ± SD from measurements of filamentous colonies in 10 biological repeats. Scale bar: 0.2 mm.

We next examined the association of Gcn5-dependent* FLO11* expression and yeast dimorphism. We performed phenotypic analysis with the same strains used for measuring* FLO11* transcripts in Figure 1(c). The* FLO11*-deletion strain (*flo11*Δ) was used as a control for complete loss of haploid invasive growth. Haploid* flo11*Δ cells were completely washed away from the surface of YPD agar medium (Figure 2(a),* flo11*Δ). The presence of a plasmid containing a functional* GCN5* in* gcn5*Δ (*gcn5*Δ +* GCN5*) restored about 40% of the cells remaining on the agar surface; less than 15% and approximately 22% cells remained in* gcn5*Δ (*gcn5*Δ + vector) and* gcn5*Δ transformed with the catalytic dead version of Gcn5 (*gcn5*Δ + E173Q), respectively (Figure 2(a)). In diploids, filamentous development was substantially impaired in* gcn5*Δ (*gcn5*Δ + vector) and E173Q (*gcn5*Δ + E173Q) strains; approximately 10~12% of the population showed pseudohyphal growth after 6 d on SLAD agar medium (Figure 2(b)). The defective phenotype was rescued in part by introducing a copy of* GCN5* to the* gcn5*Δ (*gcn5*Δ +* GCN5*). These results suggest that Gcn5 plays a regulatory role in both haploid invasive and diploid filamentous growth in yeast. Induction of* FLO11* expression by amino acid starvation (with 3AT) has distinct patterns in haploids and diploids. Gcn5 is required for optimal* FLO11* induction and its HAT activity is important for both* FLO11* expression and dimorphic growth in yeast.

### 3.3. Transcription Factor Gcn4 Binds to the* FLO11* Promoter in Response to Amino Acid Starvation

Gene-specific and time-dependent orders of events are believed to link chromatin structure modifications and transcription activation. The SAGA complex containing Gcn5 plays a key role in activating genes that allow yeast to metabolize galactose as a carbon source. Similar to carbohydrate starvation, amino acid starvation is sensed by yeast cells trough diverse pathways [21, 33].* GCN4* was identified by expression microarray analysis as a positive regulator involved in such nutrient stresses [60]. In addition to being a metabolic regulator, Gcn4 is involved in regulating* FLO11* expression [21, 61, 62]. Gcn5 was recruited to gene promoters via the transcription activator Gcn4 under conditions of amino acid starvation [51, 63]. Thus, Gcn4 is likely involved in the Gcn5-mediated* FLO11* induction under amino acid starvation condition.

**Figure 3:**
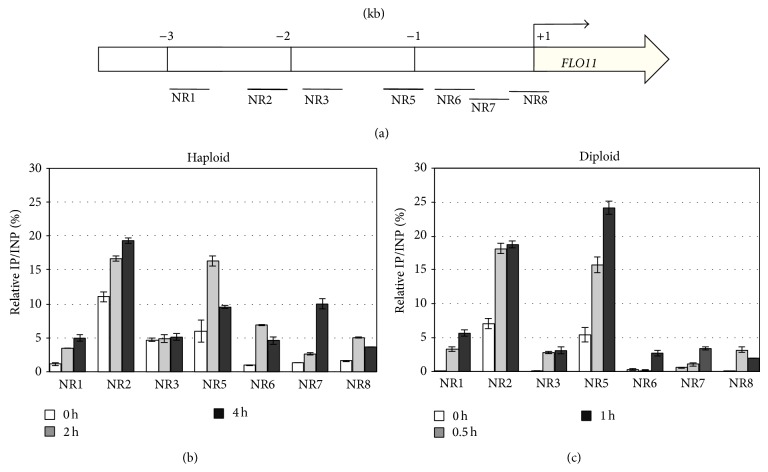
The transcription factor Gcn4 is recruited to the* FLO11* promoter during the course of 3AT induction. (a) A schematic representation of the amplicon locations at the upstream region of* FLO11* promoter with primer sets for chromatin immunoprecipitation analysis (ChIP-qPCR). +1 indicates the transcription start site of* FLO11*. Cells from haploids (b) and diploids (c) were collected at the indicated times to measure the chromatin association of Gcn4 at the* FLO11* promoter region (NR1~NR8, noncoding RNA) during 3AT induction. Association of Gcn4-Flag (a chromosome-integrated copy of* GCN4* tagged with a Flag epitope) at the NR sites was detected by ChIP and anti-Flag antibody. Data are mean ± SD from three biological repeats and normalized to input (IP/INP). The fold changes of amplified genomic DNA relative to* ACT1* are indicated (internal control of qPCR).

We postulated that the “ordered recruitment” of Gcn4-Gcn5 might take place at the* FLO11* promoter, similar to that observed at the* GAL1* and* INO1* promoters [57, 64]. We tested this possibility by using ChIP assay to analyze Gcn4 occupancy at the upstream region of the* FLO11* promoter. We designed specific primer pairs to detect the ChIP-DNA in different regions at the* FLO11* promoter (Figure 3(a)). Because of the distinct* FLO11* expression patterns shown in Figure 1(b), we performed Gcn4-ChIP experiments at 2 and 4 h for haploids (Figure 3(b)) and 0.5 and 1 h for diploids (Figure 3(c)) with 3AT treatment. In the absence of 3AT (time = 0 h), the chromatin association of Gcn4 at the* FLO11* promoter was barely detectable in diploids (Figure 3(b)) and was insignificant in haploids (Figure 3(c)) except at NR2 and NR5, which suggests a very low level of Gcn4 occupancy before 3AT induction. Gcn4 occupancy was enhanced with 3AT treatment in both haploids and diploids (Figures 3(b) and 3(c)), which indicates a direct recruitment of Gcn4 to the* FLO11* promoter at the very early stage of amino acid starvation. The most notably enhanced Gcn4 occupancy was at NR2 and NR5 in both haploids and diploids, approximately 2.2 (NR2) and 1.2 kb (NR5) upstream of the initiation start site of* FLO11* (Figure 3(a)).

Our results agree with findings that the sites for a coordinated regulation of* FLO11* transcription by Gcn4 and Hac1 are mapped to approximately 1 and 2 kb upstream of the ATG of* FLO11* by* FLO11*::*lacZ* promoter assay [61, 62]. Here, we further demonstrate the direct-chromatin association of Gcn4 to specific binding sequences at the* FLO11* promoter. Interestingly, the Gcn4 binding region overlaps with the toggle sites of Flo8 and Sfl1 at the* FLO11* promoter [43, 45], whereby Flo8 and Sfl1 reciprocally control the switches of* FLO11* transcription depending on the Rpd3L complex. Gcn4 may cooperate with Flo8 on 3AT activation to induce* FLO11* expression and supports a role of cross talk between transcription factors to restrict chromatin accessibility for activators and transcriptional machinery.

### 3.4. Gcn5 Regulates Transcription of ncRNA* ICR1* and* FLO11* in Response to Amino Acid Starvation

Two cis-interfering long noncoding RNAs encoded by* PWR1* and* ICR1* are located in the intergenic region upstream of the* FLO11* promoter [43, 45]. We have shown that Gcn5 is required for induced* FLO11* expression (Figure 1) and Gcn4 occupancy at the specific regions of the* FLO11* promoter is enriched by 3AT (Figure 3). Gcn4 has been shown to recruit Gcn5 to promoter regions of several genes involved in amino acid biosynthesis, such as* HIS3* [51], to regulate gene expression. Interestingly, the binding sites of Gcn4 at the* FLO11* promoter overlap with the toggle site of Sfl1 and Flo8 for expression of the* PWR1* and* ICR1.*

**Figure 4:**
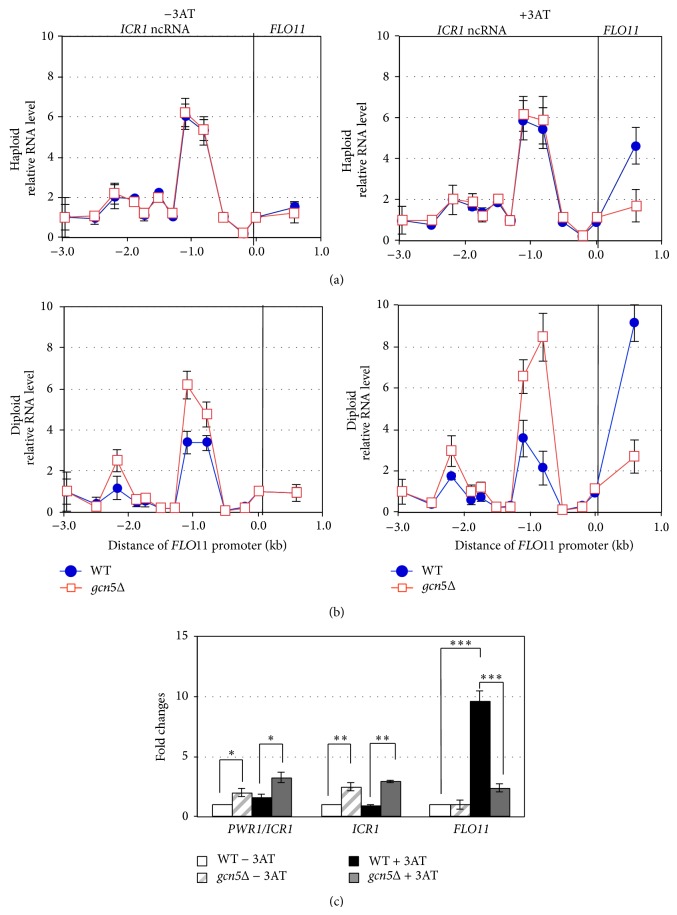
Gcn5 regulates transcription of* ICR1* and* FLO11* in response to 3AT. (a) A schematic representation of the transcription initiation sites and direction of ncRNAs* ICR1* and* PWR1* at the* FLO11* promoter (adapted from [43]). (b) RT-qPCR analysis of expression of ncRNA* ICR1* and* FLO11*. Haploid (two top panels) or diploid (two bottom panels) wild type (WT) and* gcn5*Δ strains were treated with or without 10 mM 3AT. Cells were cultured to early log phase in minimal medium and induced by 3AT for 2 h (haploids) or 1 h (diploids). In total, 12 sets of strand-specific primers tiled from −15 bp to −3 kb upstream of the* FLO11* promoter. The transcript levels were normalized to that of* ACT1.* The signal at −3 kb of the* FLO11* promoter in wild type was set to 1. (c) Quantification of transcript levels of* ICR1*,* ICR1/PWR1,* and* FLO11* in diploids treated with or without 3AT. Data are derived from Figure 4(b) (two bottom panels) and the transcript levels at specified locations are indicated in parentheses. Data are mean ± SD from 3 biological repeats. ^∗∗∗^ *P* < 1*E* − 10; ^∗∗^ *P* < 1*E* − 5; ^∗^ *P* < 1*E* − 2 by Student's *t*-test.

We hypothesized that Gcn5 is involved in transcription of the ncRNA* ICR1* to causally regulate* FLO11* expression. We examined the transcript levels of* ICR1* and* FLO11* in response to 3AT by using a set of strand-specific tiling primers spanning 3 kb upstream and 0.5 kb downstream of the transcriptional start site of* FLO11* (Figure 4(a)).* ICR1* transcript abundance was enriched at approximately 2.2 and 0.8~1.1 kb upstream of the* FLO11* transcriptional start site in both haploids and diploids (Figures 4(a) and 4(b)). These two regions with accumulated* ICR1* transcripts are fairly colocalized with the preferred Gcn4 binding sites at NR2 and NR5 (Figure 3(a)). Our results agree with previous findings [43]. Furthermore, we found an inverse association of* ICR1* and* FLO11* transcription in our assay. In haploids, the expression level and pattern of* ICR1* were nearly indistinguishable with or without 3AT and in the WT and* gcn5*Δ; however,* FLO11* was significantly induced by 3AT in the WT but not* gcn5*Δ (Figure 4(a)). Therefore, Gcn5 is essential for the 3AT-dependent* FLO11* induction and may be dispensable for* ICR1* expression in haploids. In addition,* ICR1* expression may be independent of 3AT treatment.

WT and* gcn5*Δ diploids differed in the expression of* ICR1* and* FLO11*. With and without 3AT, the* ICR1* level was about 2-fold higher in* gcn5*Δ yeast than the WT, which suggests that Gcn5 represses* ICR1* expression regardless of 3AT in diploids (Figure 4(b)). In agreement with these results (Figures 1(c) and 2(b)),* FLO11* expression was significantly induced by amino acid starvation to 10-fold in WT but only twofold in* gcn5*Δ (Figure 4(b)), so Gcn5 is the major regulator of 3AT-induced* FLO11* expression, with a minor Gcn5-independent induction in diploids. These results are consistent with the phenotypic observation in Figure 2(b) that pseudohyphal development is lost in* gcn5*Δ. Gcn5 may exert a differential control for* FLO11* induction by 3AT in haploids and diploids (Figures 4(a) and 4(b)) and the repression of* ICR1* transcription by Gcn5 in diploids may contribute to elevated* FLO11* expression.

### 3.5. Gcn5 Is a Negative Regulator of ncRNA* ICR1* Transcription in Diploid Yeast

To examine whether Gcn5 is involved in a promoter occlusion mechanism of ncRNAs to regulate* FLO11* expression in response to 3AT, we further compared the expression profiles of* PWR1, ICR1,* and* FLO11* in diploids (Figure 4(c)). In WT diploids under normal nutritional conditions, both* ICR1* and* FLO11* were expressed at low levels, whereas nutrient stress induced by 3AT greatly increased* FLO11* level, by 10-fold (*P* = 4.7*E* − 15) (Figures 4(b) and 4(c)). In* gcn5*Δ diploids, the basal levels of* ICR1* transcripts were higher than those of the WT (*P* = 2.4*E* − 9) regardless of 3AT induction (*P* = 2.3*E* − 8) (Figures 4(b) and 4(c)), so* ICR1* expression by Gcn5 is independent of 3AT. However, with 3AT,* FLO11* expression was reduced in* gcn5*Δ diploids (Figure 4(c); *P* = 2*E* − 12), so Gcn5 is a major regulator of* FLO11* induction by 3AT. Furthermore, we investigated the association of gene expression changes in ncRNAs and* FLO11* by correlation analysis. Interestingly, we found a significant inverse correlation between the expression of* ICR1* and* FLO11* in* gcn5*Δ with (*R* ^2^ = −0.4, *P* < 1*E* − 10) and without (*R* ^2^ = −0.72, *P* < 1*E* − 10) 3AT induction (Figure 4(c)). Our results suggest a role for Gcn5 in activating* FLO11* expression in response to amino acid starvation in diploids by negatively regulating the expression of ncRNA* ICR1*.

### 3.6. Cell-Type-Specific Regulation of Gcn5 on ncRNA* ICR1* Transcription

The functional link between chromatin structure and transcription processes is the “histone code,” whereby the various covalent modifications on histone tails define special patterns and affect chromatin-associated proteins at specific loci [65]. Such epigenetic controls were reported to regulate variegated* FLO11* expression [38, 45]. In this study, we explored the establishment and function of Gcn5-dependent histone acetylation in transcriptional regulation on* FLO11*. We demonstrate both common and distinct features of Gcn5-mediated transcriptional regulation of* FLO11* and ncRNA* ICR1* in haploid and diploid yeast under nutrient starvation conditions. Our findings add a new layer of transcriptional control of the regulatory circuit of* FLO11* expression in dimorphic growth in response to stress conditions.

In haploids, Gcn5 is not likely involved in the expression of ncRNAs* ICR1* and* PWR1* but is still required for induced* FLO11* expression by 3AT (Figure 4(a)). However, in diploids, Gcn5 suppresses* ICR1* expression and significantly enhances the induction of* FLO11* responding to amino acid starvation (Figure 4(b)). We propose that Gcn5 is involved in different regulatory complexes and/or mechanisms in haploids and diploids. Gcn5 may simply function as a transcriptional activator of* FLO11* in haploid yeast responding to nutrient stress. In diploids, Gcn5 may be recruited by a distinct group of transcriptional factors to modify histones for regulating the mutually exclusive transcription of ncRNAs* PWR1* and* ICR1* at the* FLO11* promoter and acts with additional unknown factors mediated by nutrient stress signal to upregulate* FLO11* expression.

In budding yeast, three cell types, including haploids of opposite mating type (*MAT*a and* MATα*) and diploids (*MAT*a/*α*), have distinct developmental phenotypes such as mating, meiosis, and budding patterns that are directly attributable to their different genotypes at the mating-type locus [66]. Mating loci (*a1* and *α2)* regulate the expression of cell-type-specific genes as well as that of the* INITIATION MEIOSIS* genes such as* IME1*,* IME2,* and* IME4*. The histone acetyltransferase Gcn5 may participate in regulating the reciprocal expression of ncRNAs* ICR1* and* PWR1* via a mating-type specific mode during amino acid starvation. To test this possibility, we analyzed the filamentous phenotype of* gcn5*Δ and the* FLO11* expression in a pseudodiploid strain (Figure 1(a)). The filamentous phenotype in the pseudodiploid was suppressed by* gcn5*Δ, but the induced* FLO11* expression by 3AT in the pseudodiploid was similar to that in haploids (data not shown), so cell-type-specific factors other than mating loci may collaborate with Gcn5 to regulate ncRNA expression.

Alternatively, chromatin structure may play a role to regulate Gcn5-associated cell-type-specific event. For example, in sporulation induced by nutrient starvation, a1/*α*2 heterodimer suppresses expression of two ncRNAs,* IRT1* (*IME1 regulatory transcript 1*) and* IME4-AS*, in diploids but not in haploids.* IRT1* is located at the* IME1* promoter and* IME4-AS* is an antisense ncRNA located at the coding region of* IME4*. In haploids,* IME4* is repressed by* IME4-AS*, and transcription of* IRT1* represses* IME1* by establishing a repressive chromatin state. In contrast, the a1/*α*2 dimer inhibits the expression of* IRT1* and* IME4-AS* in diploid cells, which then activates* IME1* and* IME4* and allows diploid cells to enter into sporulation process. Such regulation requires chromatin modifiers including Set2 (histone methyltransferase) and Set3 (histone deacetylase) complexes to establish repressive chromatin structures [67, 68]. Thus, Gcn5 may functionally associate with Set2 and Set3 to regulate ncRNAs* ICR1* and* PWR1* expression in a cell-type-specific manner during nutrient starvation.

**Figure 5:**
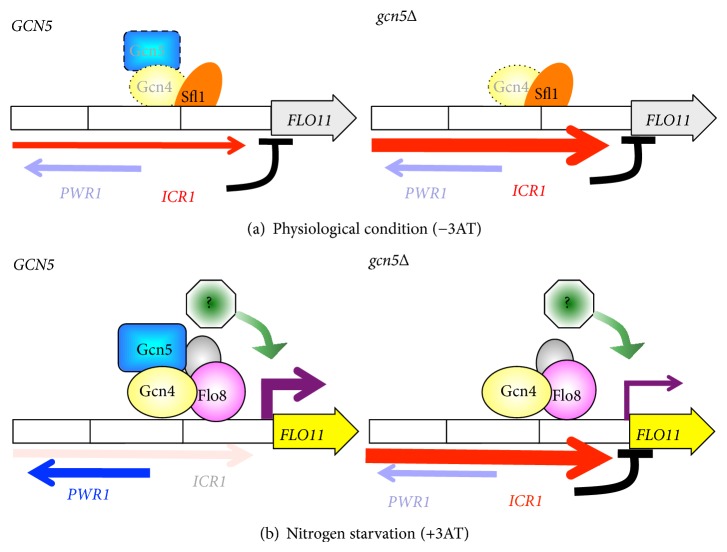
A model of Gcn5-mediated transcriptional regulation of ncRNA* ICR1* and* FLO11* in diploid yeast. (a) Under physiological conditions (−3AT), the Sfl1 (a repressor, oval in orange) associates with the* FLO11* promoter to inhibit the transcription of the ncRNA* PWR1*, which leads to enhanced transcription of another ncRNA,* ICR1*, for repressed* FLO11* transcription. The reciprocal interference between* PWR1* and* ICR1* transcription and suppression of* FLO11* expression by active* ICR1* transcription are adapted from [43]. Without 3AT induction, Gcn5 may be recruited by a marginal level of Gcn4 bound at the* FLO11* promoter (left panel). Because deletion of* GCN5* enhances* ICR1* expression twofold (right panel), Gcn5 may play a role in regulating transcription of the* PWR1* and* ICR1* independent of 3AT. However, the net transcription of* FLO11* is minimal in the WT without 3AT induction. (b) In nutrient stress such as amino acid starvation induced by 3AT (+3AT), a substantial level of Gcn4 and Gcn5 is recruited to the* FLO11* promoter. Because of overlapped binding sequences, Gcn4-Gcn5 may collaborate with Flo8 (an activator, circle in purple) to compete with Sfl1 for binding to the toggling sites and activate* PWR1* expression to block* ICR1* transcription, which then derepresses the expression of* FLO11* (left panel). Deletion of* GCN5* enhances* ICR1* expression and then reduces* FLO11* transcription (right panel). Since* gcn5*Δ diploid yeast still maintains twofold induction of* FLO11*, a Gcn5-independent factor (octagon in green) is likely involved in* FLO11* expression in response to 3AT induction.

We propose a model to hypothesize the new role of Gcn5 in regulating* FLO11* expression in diploids responding to nutrient stress (Figure 5). Under physiological conditions, the Sfl1 repressor associates at the* FLO11* promoter to inhibit transcription of the ncRNA* PWR1*, which leads to permissive transcription of the ncRNA* ICR1* [43, 45]. In both haploids and diploids, the net* FLO11* transcription remains minimal because of the negative control from the actively transcribed ncRNA* ICR1* by an antisense orientation at the promoter region of* FLO11*. DNA association of the transcription activator Gcn4 near the toggle sites of Sfl1 and Flo8 may have a limited effect on regulating transcription of the ncRNAs* PWR1* and* ICR1* probably because of low protein concentration and/or less competition for binding to DNA. Marginal upregulation of* PWR1*, probably by Gcn4-Gcn5, results in slightly reduced* ICR1* expression, which may not be sufficient to derepress* FLO11* without a stress signal (Figure 5(a)). With 3AT to induce amino acid starvation, Sfl1 is dissociated from the toggle sites, and in turn, the activator Flo8 binds to DNA and activates* PWR*1 transcription. Gcn4 recruits Gcn5 substantially to the Flo8-Sfl1 toggling sites to further activate* PWR1* leading to suppressing* ICR1*, which resets the transcriptional module for activation of* FLO11* responding to nutrient stress (Figure 5(b)). A Gcn5-independent factor may be required for the full induction of* FLO11*. Our model presents Gcn4-Gcn5 as new regulators involved in the* FLO11* induction in diploid yeast responding to nutrient stress by participating in the coordinated regulatory circuit consisting of Sfl1, Flo8, Rpd3L-containing complex, and the ncRNAs* ICR1* and* PWR1*.

## 4. Conclusions

This study reveals that the histone acetyltransferase Gcn5 is required for pseudohyphal development in the budding yeast* S. cerevisiae*. We found distinct expression patterns of* FLO11* in haploid and diploid yeast responding to amino acid starvation induced by 3AT. Deletion of* FLO11* or* GCN5* resulted in loss of invasive growth in haploids and filamentous formation in diploids. ChIP analysis revealed for the first time an association of Gcn4 with the* FLO11* promoter region that overlaps with the binding sites of Sfl1 and Flo8 at a critical location to mediate the mutually exclusive transcription of two long ncRNAs,* ICR1* and* PWR1*. Gcn5 is required for full induction of* FLO11* expression in diploid yeast under nutrient stress conditions. Finally, Gcn5 inhibits transcription of* ICR1*, which then depresses* FLO11* for basal and induced expression. Our research on the mechanistic role of Gcn5 in regulating* FLO11* expression and pseudohyphal development suggests potential therapeutic targets for treatment of fungal diseases.

## Figures and Tables

**Table 1:** Yeast strains used in this study.

Strain	Genotype	Reference
SLY16	*MATa, ura3–52 leu2*∷*hisG his3*∷*hisG trp1*∷*hisG *	Laboratory of G. Fink

SLY17	*MATα, ura3–52 leu2*∷*hisG his3*∷*hisG trp1*∷*hisG *	Laboratory of G. Fink

SLY134	*MATa, ura3–52 leu2*∷*hisG his3*∷*hisG flo11*Δ∷*HIS3 *	[39]

SLY506	*MATa, ura3–52 leu2*∷*hisG his3*∷*hisG trp1*∷*hsiG gcn5*Δ∷*HIS3 *	This study

SLY507	*MATa, ura3–52 leu2*∷*hisG his3*∷*hisG trp1*∷*hsiG snf1*Δ∷*TRP1 *	This study

SLY549	*MATα, ura3–52 leu2*∷*hisG his3*∷*hisG trp1*∷*hsiG gcn5*Δ∷*HIS3 *	This study

SLY551	*MATa, ura3–52 leu2*∷*hisG his3*∷*hisG trp1*∷*hsiG gcn5*Δ∷*HIS3 + *[*pRS316-GCN5*]	This study

SLY553	*MATa, ura3–52 leu2*∷*hisG his3*∷*hisG trp1*∷*hsiG gcn5*Δ∷*HIS3 + *[*pRS316*]	This study

SLY555	*MATa, ura3–52 leu2*∷*hisG his3*∷*hisG trp1*∷*hsiG gcn5*Δ∷*HIS3 + *[*pRS316-gcn5-E173Q*]	This study

SLY565	*MATa, ura3–52 leu2*∷*hisG his3*∷*hisG trp1*∷*hsiG * + [pRS415-*MATα*2]	This study

SLY569	*MATα, ura3–52 leu2*∷*hisG his3*∷*hisG trp1*∷*hsiG * + [pRS315-*MAT*a1]	This study

SLY570	*MATα, ura3–52 leu2*∷*hisG his3*∷*hisG trp1*∷*hsiG gcn5*Δ∷*HIS3 * + [pRS315-*MAT*a1]	This study

SLY594	*MATa, ura3–52 leu2*∷*hisG his3*∷*hisG trp1*∷*hsiG gcn5*Δ∷*HIS3 * + [pRS415-*MATα*2]	This study

SLY678	*MATα, ura3–52 leu2*∷*hisG trp1*∷*hisG GCN4-2Flag*∷*TRP1 *	This study

SLY848	MATa/*α*, *ura3–52/ura3–52 leu2*∷*hisG/leu2*∷*hisGhis3*∷*hisG/HIS3 GCN4-2Flag*∷*TRP1/trp1*∷*hisG *	This study

SLY1062	MATa/*α*, *ura3–52/ura3–52leu2*∷*hisG/leu2*∷*hisGhis3*∷*hisG/his3*∷*hisG trp1*∷*hisG/trp1*∷*hisGgcn5*Δ∷*HIS3/gcn5*Δ∷*HIS3 + *[*pRS316-GCN5*]	This study

SLY1063	MATa/*α*, *ura3–52/ura3–52leu2*∷*hisG/leu2*∷*hisGhis3*∷*hisG/his3*∷*hisG trp1*∷*hisG/trp1*∷*hisGgcn5*Δ∷*HIS3/gcn5*Δ∷*HIS3 + *[*pRS316*]	This study

SLY1064	MATa/*α*, *ura3–52/ura3–52leu2*∷*hisG/leu2*∷*hisGhis3*∷*hisG/his3*∷*hisG trp1*∷*hisG/trp1*∷*hisGgcn5*Δ∷*HIS3/gcn5*Δ∷*HIS3 + *[*pRS316-gcn5-E173Q*]	This study
